# Whole-Transcriptome Sequencing Integrative Analyses Reveal Expression Profiles and ceRNA Regulatory Network of Huoyan Goose Egg Production

**DOI:** 10.3390/ani16071053

**Published:** 2026-03-30

**Authors:** Yibing Liu, Kun Wang, Xinxin Zhang, Ning Yu, Wenjing Ma, Yunwei Fan, Hui Zhao

**Affiliations:** 1 Liaoning Academy of Agricultural Sciences, No. 84 Dongling Road, Shenyang 110161, China; liuyb_0423@163.com (Y.L.); rain0609123@126.com (N.Y.); 2 College of Animal Science and Veterinary Medicine, Shenyang Agricultural University, Shenyang 110866, China; syauwk1618@163.com (K.W.); zzyzxx030416@163.com (X.Z.); mwj010707@163.com (W.M.); 15566332389@163.com (Y.F.)

**Keywords:** Huoyan goose, ovary, egg production performance, whole-transcriptome sequencing, ceRNA regulatory network

## Abstract

According to statistics from the FAO, China is the country with the largest number of geese raised and slaughtered worldwide. The goose farming industry is a traditional sector of Chinese livestock production. Huoyan goose is a unique local breed in China, originating from Changtu County in Liaoning Province, with excellent egg production performance. We collected ovarian stroma tissue samples of Huoyan geese at four different stages, from the pre- to post-egg-laying period, with a sample size of five for each group. Using whole-transcriptome sequencing, we identified a total of 13,193 genes, 2814 lncRNAs, and 202 miRNAs, of which 2112 genes, 187 lncRNAs, and 37 miRNAs were differentially expressed between the groups. GO and KEGG functional enrichment results indicated that these genes and non-coding RNAs were involved in multiple pathways related to egg-laying and ovarian development, including the ‘PI3K-Akt signaling pathway’ and the ‘ovulation cycle’. After predicting the target relationships among differentially expressed genes, lncRNAs, and miRNAs, we constructed a competitive endogenous RNA network that could regulate egg production. Our results provide further insights into the regulatory roles of non-coding RNAs in egg production and ovarian development, and offer new references for improving egg production performance of geese.

## Simple Summary

The Huoyan goose is a small-sized local breed endemic to China and is characterized by excellent egg production performance. Egg-laying traits are key production and economic traits in geese, and are regulated by genetics and the environment. In the present study, we investigated the genetic regulation of egg-laying traits in Huoyan geese. We collected the ovarian stroma tissues of Huoyan geese in pre-, early-, peak-, and post-egg-laying periods and performed whole-transcriptome sequencing. We generated expression profiles of mRNAs, lncRNAs, and miRNAs in different egg-laying periods, and identified key factors through functional enrichment analysis and targeted relationship prediction. We constructed a competitive endogenous RNA network, consisting of six lncRNAs, five miRNAs, and nine genes, related to the regulation of ovarian development and the egg production process in Huoyan geese. This research provides a new reference for exploring the molecular regulatory mechanisms of egg production performance and for improving the egg-laying traits of geese.

## 1. Introduction

China is a major global poultry producer and has rich genetic resources for geese, with many local and hybrid breeds widely distributed in various regions [1,2]. The Huoyan goose is a small-sized breed of Chinese white geese originating from Changtu County in Liaoning Province, and it is known for its excellent egg production performance [3]. Under suitable breeding and management conditions, the annual egg production of Huoyan geese has exceeded 100 eggs. As an important poultry species, the egg production performance of geese affects the economic benefits of farmers and businesses. With the development of the geese industry, improving the egg-laying traits has become a key research concern.

Egg-laying traits are polygenic traits that are regulated by genetic and environmental factors and have relatively low heritability [4]. The egg-laying process is regulated by hormone levels in the body, such as gonadotropin-releasing hormone (GnRH), prolactin (PRL), follicle-stimulating hormone (FSH), and luteinizing hormone (LH) [5]. It also involves physiological processes, including energy metabolism, protein synthesis, and storage, and requires coordination and cooperation among multiple organs within the organism, such as the hypothalamus, pituitary, and ovary [6]. Because egg-laying traits are shaped by many genetic and environmental factors, accurately pinpointing the major drivers is essential for accelerating genetic improvement.

Based on whole-transcriptome sequencing data, mRNAs and non-coding RNAs (ncRNAs) under specific conditions were determined. Competitive endogenous RNA (ceRNA) is a molecular regulatory mechanism in which ncRNAs—such as lncRNAs and circRNAs—and protein-coding genes competitively bind to miRNAs via miRNA response elements (MREs) to release the inhibitory effects of miRNAs on their target genes [7]. The ceRNA regulatory networks play crucial roles in various biological processes [8,9]. In recent years, an increasing number of studies have been conducted to explore the egg production traits of poultry using transcriptome data, and preliminary progress has been made, particularly in chickens and ducks. Li et al. [10] performed a comparative analysis of transcriptome sequencing data from the pituitary of Hy-Line Brown hens at different growth stages. They identified a ceRNA network associated with developmental and reproductive processes in hens, containing 427 lncRNA–miRNA–mRNA pairs. Zhang et al. [11] collected ovaries from low- and high-yield Gaoyou ducks for RNA-seq. They detected 72 mRNAs, 23 lncRNAs, and four circRNAs that were differentially expressed between the two groups, which may regulate the ovarian function of ducks through ovarian steroidogenesis and tryptophan metabolism signaling pathways. However, further studies are required to investigate the role of ceRNAs in the regulation of goose egg production and ovarian development.

In this study, we focused on Huoyan geese, and ovaries at different egg-laying stages were collected for whole-transcriptome sequencing. By comparing the differences at the transcriptome level among the groups, we profiled lncRNA, miRNA, and mRNA expression in the Huoyan geese ovaries. Furthermore, we constructed a competing endogenous RNA regulatory network associated with goose egg production and ovarian development. Our results preliminarily revealed the molecular regulatory mechanism of egg-laying traits in Huoyan geese to provide new references for the genetic improvement of egg production performance in geese.

## 2. Materials and Methods

### 2.1. Experimental Animals and Sample Collection

Huoyan geese were provided by the Haicheng Jiarong Agricultural Technology Co., Ltd. Breeding Farm (Anshan, China). All individuals were raised under the same environmental conditions, with free access to food and water. Huoyan geese reach sexual maturity at around five months of age and can commence laying at six months. Based on the age and physiological status, we selected four stages for sampling: the pre-egg-laying period (150 d of age, PR group), early-egg-laying period (180 d of age, EA group), peak-egg-laying period (270 d of age, PE group), and post-egg-laying period (400 d of age, PO group). PR and PO are non-egg-laying periods, while EA and PE are egg-laying periods. At each stage, five Huoyan geese were randomly selected. All geese were euthanized by neck exsanguination following anesthesia, and intact ovaries were rapidly collected. Then the follicles were removed, and the ovarian stroma tissues were frozen in liquid nitrogen for later use. Total RNA was extracted from each sample using TRIzol reagent (TianGen, Beijing, China). To ensure the quality of the total RNA, we used the NanoDrop 2000 spectrophotometer (Thermo Fisher Scienrific, Waltham, MA, USA) to detect RNA purity and concentration, and the Agilent 2100 Bioanalyzer System (Agilent Technologies, Inc., Santa Clara, CA, USA) was used to detect RNA integrity.

### 2.2. Library Construction and RNA Sequencing

cDNA libraries of lncRNAs and small RNAs were generated separately. For mRNAs and lncRNAs, the Ribo-Zero rRNA Removal Kit (Epicentre, Madison, WI, USA) was used to remove rRNA from total RNA. Strand-specific libraries were generated using the NEBNext^®^ Ultra^TM^ Directional RNA Library Prep Kit (NEB, Beverly, MA, USA), following the manufacturer’s recommendations. For small RNAs, libraries were generated using the TruSeq^TM^ Small RNA Sample Prep Kit (Illumina, San Diego, CA, USA). The AMPure XP system was used to purify PCR products. After the library quality assessment was qualified, the Illumina NovaSeq6000 platform (Illumina, San Diego, CA, USA) was used for whole-transcriptome sequencing, generating 150 bp pair-end reads (mRNAs and lncRNAs) and 50 bp single-end reads (miRNAs).

### 2.3. Sequencing Data Quality Control and Analysis

For mRNAs and lncRNAs, clean data were obtained after removing adapters, reads containing more than 10% ‘N’ bases, and low-quality reads from raw data using Trimmomatic (v0.39) [12]. The goose reference genome (Anser cygnoides, GCF_002166845.1) and its annotation files were downloaded from the NCBI database (https://ftp.ncbi.nlm.nih.gov/). Mapped reads were obtained by aligning clean reads with the reference genome using HISAT2 (v2.0.4) [13]. We used StringTie (v1.3.1) [14] to assemble the mapped reads and reconstruct transcripts for subsequent analyses.

For miRNAs, Cutadapt (v3.4) [15] was used to filter the raw data, including removing adapters, reads containing more than 10% ‘N’ bases, reads with lengths < 18 nt or >30 nt, and low-quality reads. Using Bowtie tools (v1.0.0) [16], clean reads were aligned with multiple RNA databases (Silva, GtRNAdb, Rfam, and Repbase) to filter out other ncRNAs and repeats, and unannotated reads were obtained. We then mapped the unannotated reads to the reference genome to obtain the mapped reads.

### 2.4. Identification of lncRNAs and miRNAs

After genome alignment and transcript annotation, we selected lncRNAs based on the following criteria: ① transcript class_code as ‘i’, ‘x’, ‘u’, ‘o’, and ‘j’; ② exon number ≥ 2; ③ transcript length greater than 200 nt. Subsequently, we used assessment tools CPC2 (v2) [17], CPAT (v1.2.4) [18], and CNCI (v2) [19] to predict the coding potential of transcripts, considering the intersection of the results as the predicted lncRNAs.

The mapped reads of small RNA sequencing were aligned with miRbase (http://www.mirbase.org/), and the identified reads were considered known miRNAs. We used miRDeep2 (v2.0.5) [20] to predict novel miRNAs based on precursor sequence distribution, miRNA biological characteristics, and secondary structure energy.

### 2.5. Differentially Expressed Analysis of Genes, lncRNAs, and miRNAs

The raw read counts were used to perform differential expression analyses among the four comparison groups (PE vs. PR, PE vs. EA, PE vs. PO, and EA vs. PR), using DESeq2 (v1.30.1) [21]. We selected |fold change| ≥ 1.5 and *p* value < 0.01 as the criteria for screening differentially expressed genes (DEGs), differentially expressed lncRNAs (DELs), and differentially expressed miRNAs (DEMs). The FPKM (Fragments Per Kilobase of transcript per Million fragments mapped) was applied for standardization, as an indicator to measure the expression levels of genes and lncRNAs. The TPM (Transcripts Per Million) algorithm was used to normalize the expression of miRNAs. The normalized expression values were used for visualization.

### 2.6. Target Gene Prediction of lncRNAs and miRNAs

We used Bedtools (v2.25.0) [22] to select genes within 100 kb upstream or downstream of the lncRNAs as cis-regulated target genes. The correlation between the expression of lncRNAs and genes was assessed by Pearson’s correlation analysis. We selected genes with |Pearson’s *r*| > 0.9 and *p* value < 0.01 as trans-regulated target genes of lncRNAs. Based on the miRNA sequence information and binding site sequence, target gene prediction was performed using miRanda (v3.3a) [23] and TargetScan (v5.0) [24], with the criteria of binding sites > 7 and mfe < −30 kcal/mol.

### 2.7. Gene Ontology and Kyoto Encyclopedia of Genes and Genomes Analyses

ClusterProfiler (v4.4.4) [25] and KOBAS (v3.0) [26] were used to perform GO functional and KEGG pathway enrichment analyses, respectively. Functional enrichment analyses were performed on the DEGs and target genes of the DELs and DEMs. GO terms and KEGG pathways with a *p* value < 0.05 were considered significantly enriched.

### 2.8. Construction of ceRNA Regulatory Network

TargetScan (v5.0) and miRanda (v3.3a) software were also used to predict the target relationships between differentially expressed miRNAs and lncRNAs based on sequence complementarity matching and thermodynamic stability evaluation. We constructed a competing endogenous RNA network incorporating the associated target genes, and visualized the network using Cytoscape (v3.7.1) [27].

### 2.9. Quantitative Real-Time PCR Validation of RNA Sequencing Results

The extraction method for total RNA was the same as previously described in Section 2.1. We used a PrimeScript™ RT reagent kit with gDNA eraser (Takara, Dalian, China) to reverse-transcribe cDNA from total RNA according to the instructions. SYBR Green I Master Mix was used to perform qRT-PCR on the Light Cycler^®^ 480 Real-Time PCR System (Roche, Basel, Switzerland). *GAPDH* was used as the internal reference for genes and lncRNAs, and U6 was used as the internal reference for miRNAs. All the primer sequences are listed in Table S1. Three replicates were performed for each experiment. The average Ct value and the 2^−∆∆Ct^ method were employed to calculate the relative expression levels.

## 3. Results

### 3.1. Overview of RNA Sequencing

For the strand-specific RNA sequencing data, after quality control of 20 samples, we obtained 15.14 to 18.07 Gb of clean data. The GC content was between 44.42% and 46.17%, and the Q30 values ranged from 90.59% to 94.47%. For individual samples, over 101,340,730 clean reads remained after screening, and over 94,861,729 mapped reads were obtained, with a mapped ratio exceeding 92.74% (Table S2). Small RNA sequencing generated a total of 9,871,202 to 16,581,807 raw reads for individual samples. The Q30 value exceeded 94.53%. After quality control, the clean reads of the 20 samples ranged from 9,541,212 to 15,620,171 (Table S3). The sequencing data were of sufficient quality for subsequent analysis.

### 3.2. Characteristics of mRNAs, lncRNAs, and miRNAs

**Figure 1:**
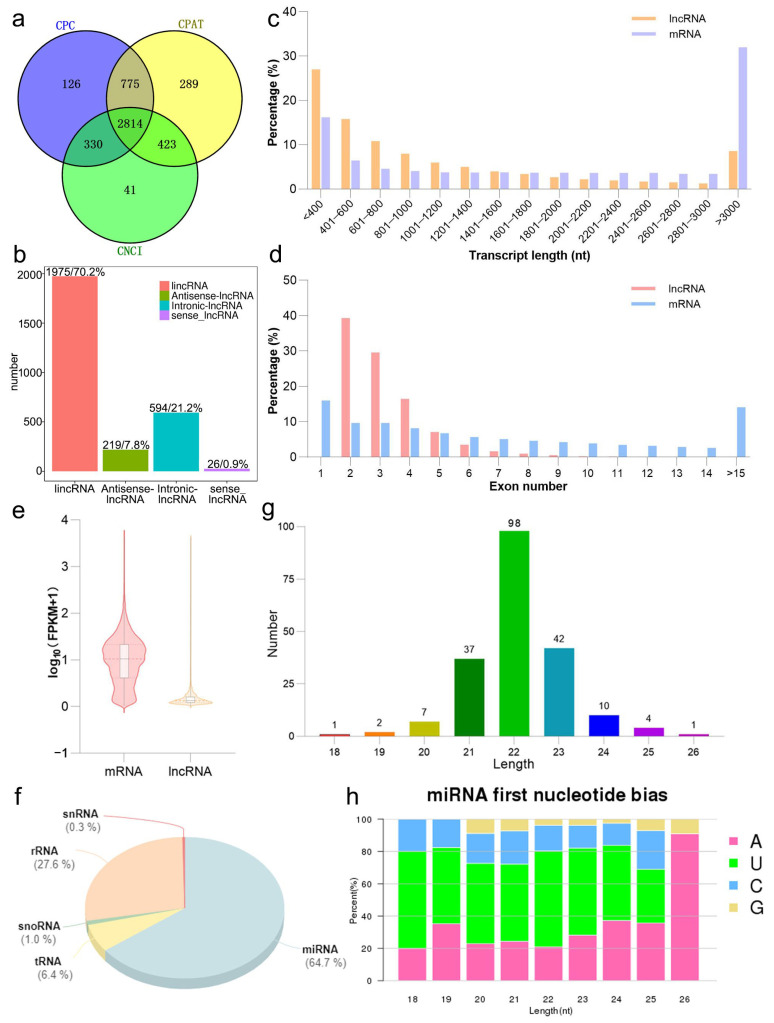
Characteristics of mRNAs, lncRNAs, and miRNAs: (**a**) Venn diagram of identified lncRNAs from CPC, CPAT, and CNCI; (**b**) classification of putative lncRNAs; (**c**) transcript length distribution of mRNAs and lncRNAs; (**d**) exon number distribution of mRNAs and lncRNAs; (**e**) comparison of expression levels of mRNAs and lncRNAs, the value of log10(FPKM + 1) is plotted on the *Y*-axis; (**f**) classification of miRNA types; (**g**) length distribution of miRNAs; (**h**) the base bias analysis of miRNAs.

Through strand-specific RNA sequencing, 13,193 genes were identified after transcript assembly and genome annotation. We screened 2814 lncRNAs following strict criteria (Figure 1a), including 1975 lincRNAs (70.2%), 219 antisense lncRNAs (7.8%), 594 intronic lncRNAs (21.1%), and 26 sense lncRNAs (0.9%) (Figure 1b). Distribution diagrams of transcript lengths and exon numbers are shown in Figure 1c,d. The lncRNAs with a length of less than 400 nt and containing two exons accounted for a higher proportion of total lncRNAs. Meanwhile, most mRNAs were longer than 3000 nt and contained one exon. By further comparing the expression levels of the mRNAs and lncRNAs, we observed that the expression levels of the lncRNAs were significantly lower than those of the mRNAs (Figure 1e). For small RNA sequencing, the sequences were categorized and annotated (Figure 1f). The results showed that 64.7% of the annotated sequences were miRNAs and 27.6% were ribosomal RNA (rRNA). These two types of small RNAs accounted for over 90% of all the annotated sequences. In total, 202 miRNAs were identified. Their length distribution is shown in Figure 1g, with most being 22 nt long. The first nucleotides of these miRNAs showed a strong preference for uracil (Figure 1h).

### 3.3. Identification of DEGs, DELs, and DEMs

**Figure 2:**
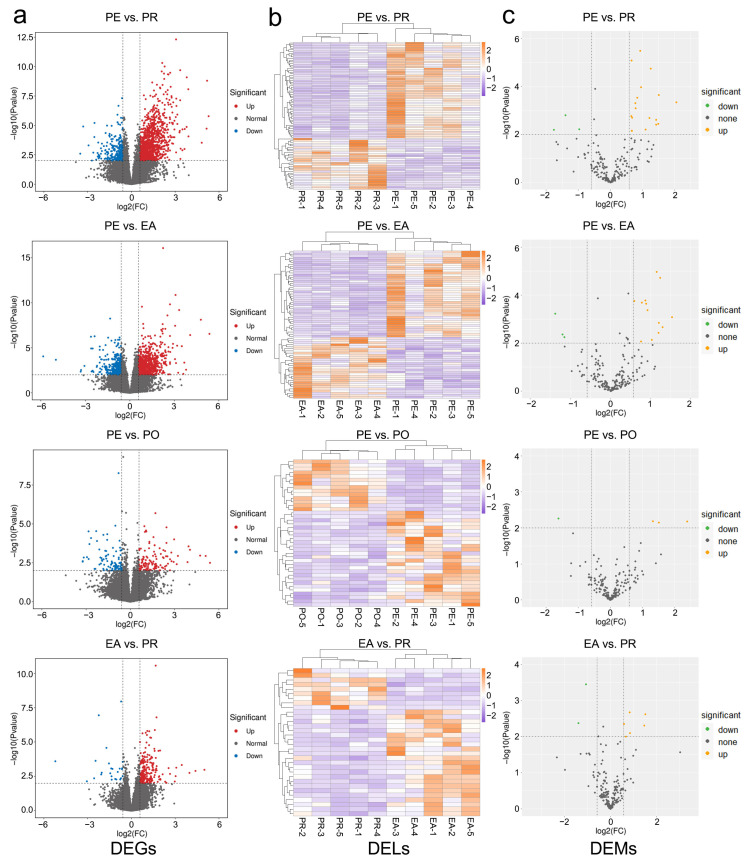
Expression of differentially expressed genes, lncRNAs, and miRNAs: (**a**) Volcano plot of DEGs. The value of −log10(*p*-value) is plotted on the *X*-axis, and the value of log2(FC) is plotted on the *Y*-axis. The two threshold lines show the standard of *p*-value = 0.01 and FC = 1.5. (**b**) Heatmap of DELs. The color scale represents the FPKM values. (**c**) Volcano plot of DEMs.

After differential expression analysis, we identified 2112 DEGs, 187 DELs, and 37 DEMs from the four comparison groups (Tables S4–S6). Of these, 1405, 1112, 196, and 238 DEGs, 86, 70, 40, and 32 DElncRNAs, and 22, 19, 4, and 8 DEmiRNAs were identified between PE and PR, between PE and EA, between PE and PO, and between EA and PR, respectively (Figure 2). The numbers of upregulated and downregulated DEGs, DELs, and DEMs are shown in Table 1. We found that the expression levels of five genes (*FHL2*, *KCNJ8*, *MYH11*, *OGN*, and *SMTN*) and one miRNA (miR-1a-3p) were significantly upregulated in all four comparison groups. In addition, 780 DEGs, 40 DELs, and 13 DEMs exhibited significant differential expression and consistent trends across two or more comparison groups.

### 3.4. Target Gene Prediction of DELs and DEMs

Subsequently, we conducted target gene prediction for the DELs and DEMs. Based on the positional relationship between genes and DELs, we identified 892 cis-regulated target genes of 187 DELs, of which 103 genes were identified as DEGs. Moreover, we identified 2313 trans-regulated target genes of DELs through expression-level correlation analysis, of which 683 were differentially expressed among the groups (Table S7). Using miRanda and TargetScan, 2536 target genes regulated by 37 DEMs were identified, of which 350 genes overlapped with the DEGs (Table S8).

### 3.5. GO and KEGG Functional Enrichment Analyses

**Figure 3:**
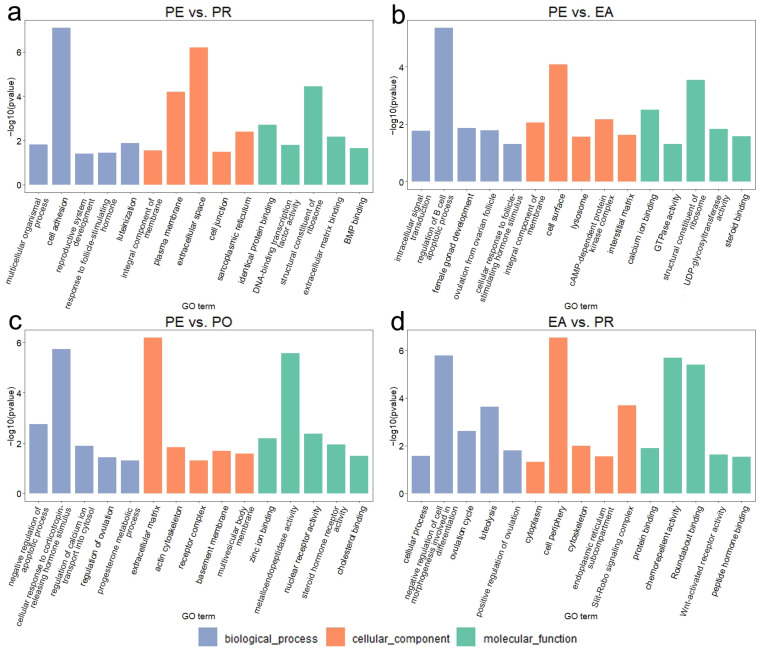
GO functional enrichment results of DEGs and target genes of DELs and DEMs in the four comparison groups: (**a**) GO results of PE vs. PR groups. (**b**) GO results of PE vs. EA groups. (**c**) GO results of PE vs. PO groups. (**d**) GO results of EA vs. PR groups.

**Figure 4:**
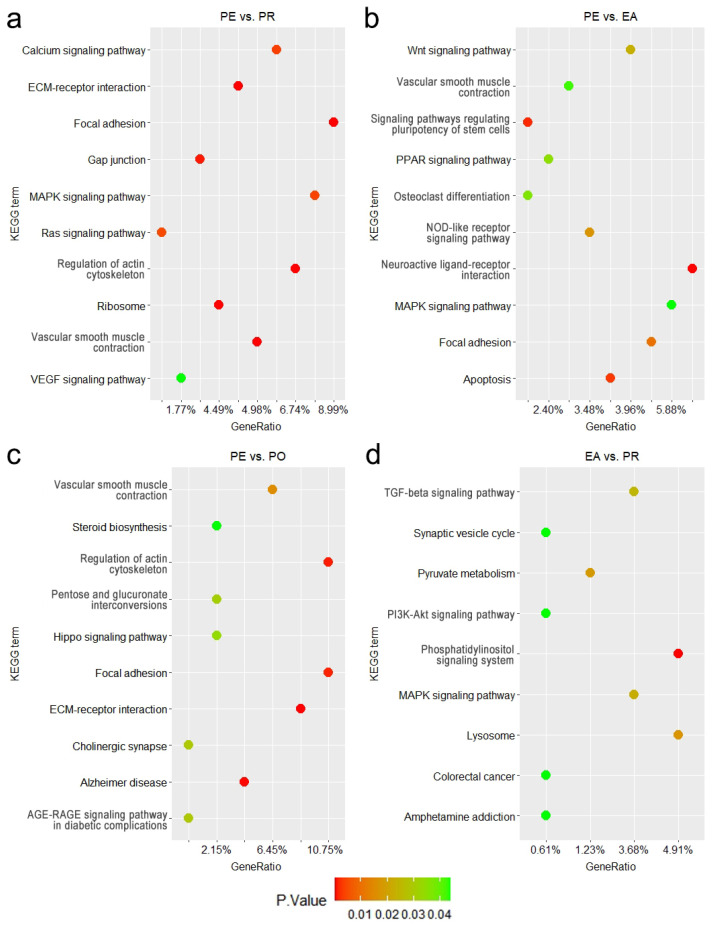
KEGG functional enrichment results of DEGs and target genes of DELs and DEMs in the four comparison groups: (**a**) KEGG results of PE vs. PR groups. (**b**) KEGG results of PE vs. EA groups. (**c**) KEGG results of PE vs. PO groups. (**d**) KEGG results of EA vs. PR groups.

To better understand the potential functions of the key genes and ncRNAs, we combined the DEGs and target genes of the DELs and DEMs, then performed functional enrichment analysis. The results of GO and KEGG analyses are shown in Figure 3 and Figure 4 and Tables S9 and S10. These enriched pathways and genes within them may play a role in the process of egg production and ovarian development.

Between the PE and PR groups, 1193 GO terms were significantly enriched (Figure 3a), including 967 biological processes (BPs), 84 cellular components (CCs), and 142 molecular functions (MFs). The results showed that ‘integral component of membrane’ enriched the most genes, with ‘cell adhesion’ being the most significantly enriched term. The critical GO term ‘response to follicle-stimulating hormone’ was also significantly enriched. Based on the KEGG enrichment analysis (Figure 4a), these genes exhibited enrichment in 191 pathways, with 16 pathways showing significant enrichment, including ‘Calcium signaling pathway’ and ‘MAPK signaling pathway’. The most significantly enriched pathway was ‘focal adhesion’.

Between the PE and EA groups, 582 GO terms were significantly enriched (Figure 3b), including 448 BP terms, 58 CC terms, and 76 MF terms. The results showed that ‘integral component of membrane’ enriched the most genes, with ‘cell surface’ being the most significantly enriched term. The critical GO term ‘ovulation from ovarian follicle’ was also significantly enriched. Based on KEGG enrichment analysis (Figure 4b), these genes exhibited enrichment in 184 pathways, with 25 pathways showing significant enrichment, including the ‘PPAR signaling pathway’ and ‘WNT signaling pathway’. The most significantly enriched pathway was ‘neuroactive ligand-receptor interaction’.

Between the PE and PO groups, 361 GO terms were significantly enriched (Figure 3c), including 278 BP terms, 34 CC terms, and 49 MF terms. The results showed that ‘zinc ion binding’ enriched the most genes, with ‘extracellular matrix’ being the most significantly enriched term. The critical GO term ‘regulation of ovulation’ was also significantly enriched. Based on the KEGG enrichment analysis (Figure 4c), these genes exhibited enrichment in 96 pathways, with 13 pathways showing significant enrichment, including the ‘Hippo signaling pathway’ and ‘steroid biosynthesis’. The most significantly enriched pathway was ‘ECM-receptor interaction’.

Between the EA and PR groups, 868 GO terms were significantly enriched (Figure 3d), including 684 BP terms, 59 CC terms, and 125 MF terms. The results showed that ‘cellular process’ enriched the most genes, with ‘cell periphery’ being the most significantly enriched term. The critical GO term ’ovulation cycle’ was also significantly enriched. Based on KEGG enrichment analysis (Figure 4d), these genes exhibited enrichment in 89 pathways, with nine pathways showing significant enrichment, including the ‘TGF-beta signaling pathway’ and ‘PI3K-Akt signaling pathway’. The most significantly enriched pathway was the ‘phosphatidylinositol signaling system’.

### 3.6. Construction of the ceRNA Network

**Figure 5:**
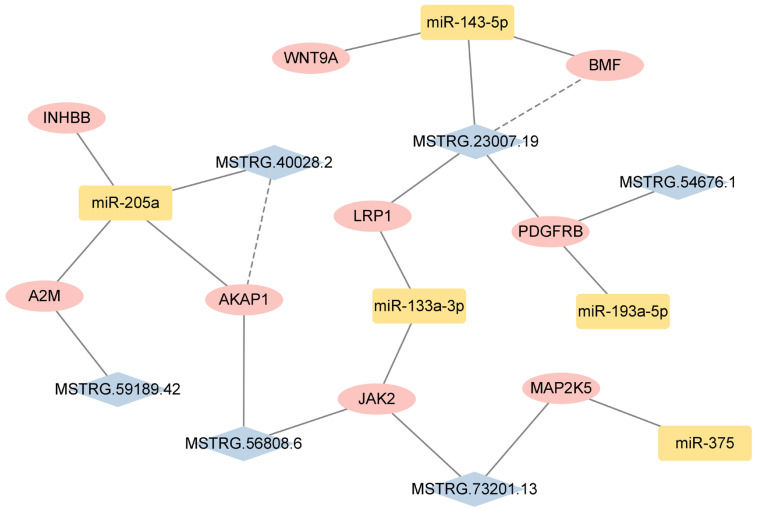
Competing endogenous RNA network of lncRNAs, miRNAs, and genes related to egg production performance. Pink circles represent mRNAs, yellow squares represent miRNAs, and blue diamonds represent lncRNAs. Solid lines indicate that non-coding RNAs have regulatory effects on the genes. Dashed lines indicate that lncRNAs as ceRNAs regulate target genes by competitively binding to miRNAs.

To examine the global regulatory relationship between genes and ncRNAs, we integrated all the DEGs, DELs, and DEMs. Subsequently, we predicted the target relationship between the DEMs and DELs based on sequence complementarity and binding stability. Combining the results of the target gene prediction, we constructed a ceRNA regulatory network involved in goose egg production performance, composed of six lncRNAs, five miRNAs, and nine genes (Figure 5). Within this regulatory network, we found that two lncRNA–miRNA–mRNA pairs, such as both lncRNA MSTRG.23007.19 and its target gene *BMF*, could be regulated by miR-143-5p. The MSTRG.40028.2/miR-205a/*AKAP1* axis was also targeted in the same way.

### 3.7. qRT-PCR Validation of Gene Expression

**Figure 6:**
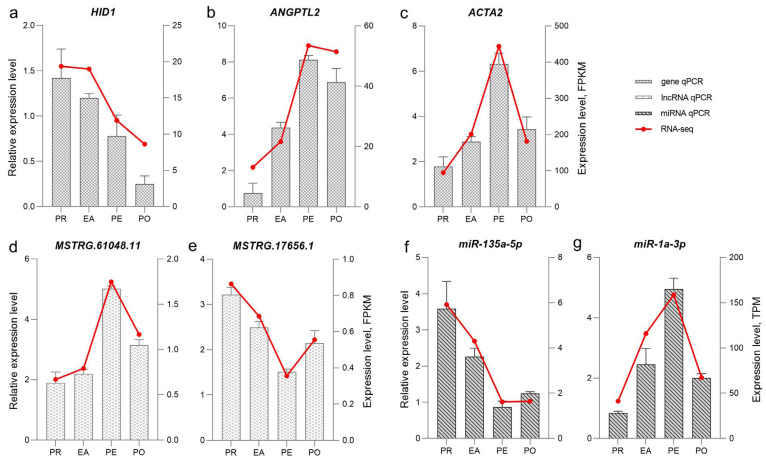
Quantitative real-time PCR validation of DEGs, DELs, and DEMs: (**a**) qRT-PCR of gene *HID1*. (**b**) qRT-PCR of gene *ANGPTL2*. (**c**) qRT-PCR of gene *ACTA2*. (**d**) qRT-PCR of lncRNA MSTRG.61048.11. (**e**) qRT-PCR of lncRNA MSTRG.17656.1. (**f**) qRT-PCR of miR-135a-5p. (**g**) qRT-PCR of miR-1a-3p.

To verify the accuracy of the RNA-seq results, we randomly selected three genes (*HID1*, *ANGPTL2,* and *ACTA2*), two lncRNAs (MSTRG.61048.11 and MSTRG.17656.1), and two miRNAs (miR-135a-5p and miR-1a-3p) that were differentially expressed between comparison groups for qRT-PCR experiments. The results are shown in Figure 6. The changes in the expression trend of the same gene in the qRT-PCR and RNA sequencing were consistent, confirming the accuracy and reliability of the RNA-seq results.

## 4. Discussion

Egg-laying traits are important economic traits in poultry production. Poultry eggs, which contain abundant nutrients, serve as an important source of high-quality protein for the human diet. Studies have shown that goose eggs contain higher levels of crude fat and fatty acids, and a richer content of minerals and vitamins, compared to chicken eggs [28,29]. The ovary is a dynamically developing reproductive organ that determines the efficiency and sustainability of egg production. At different stages of egg production, there are significant differences in ovarian structure and function [30,31].

In this study, we focused on the ovaries of Huoyan geese at different egg-laying stages and determined the expression profiles of genes, lncRNAs, and miRNAs through whole-transcriptome sequencing. We identified 13,193 genes, 2814 lncRNAs, and 202 miRNAs, of which 2112 genes, 187 lncRNAs, and 37 miRNAs were differentially expressed among the four comparison groups. The highest number of differentially expressed genes was observed in the PE vs. PR comparison, with 1405 DEGs, 86 DELs, and 22 DEMs. The reason for this difference may be that the ovarian physiological status [32] and functions [33] of the geese exhibited the most significant differences between the two stages, from sexual immaturity to peak production. In the pre-egg-laying period, the ovarian stroma lays the foundation for follicular recruitment and initial development. However, during the peak-egg-laying period, its primary role is to sustain follicle maturation, ovulation, and egg formation [34,35]. The gene expression patterns in the two stages are different, and the expression of non-coding RNAs will also change accordingly to meet the target gene regulatory requirements.

The process of egg production constitutes a multi-stage physiological cascade comprising reproductive tract development and maturation, follicular recruitment and growth, ovulation, egg formation, and expulsion. This cascade is precisely regulated by the hypothalamic–pituitary–ovarian (HPO) axis of the neuroendocrine system [36]. First, the hypothalamus secretes GnRH, which stimulates the pituitary gland to release FSH and LH, directly acting on the ovaries. FSH drives the development of pre-hierarchical follicles, and LH surges induce ovulation [37]. Yolk deposition, albumen secretion, and shell calcification occur sequentially. At last, oviposition is executed through the smooth muscle contractions [38]. In the present study, many genes and pathways were involved in this physiological cascade.

We performed GO and KEGG functional enrichment analyses on differentially expressed genes and target genes of DELs and DEMs and obtained pathways closely related to ovarian follicle development and egg production. In the GO enrichment analyses, the GO term ‘ovulation cycle’ of biological processes was significantly enriched. The ‘ovulation cycle’ is a core process of egg production in poultry. It coordinates the complete reproductive cycle through follicle development, hierarchical recruitment, ovulation, and post-ovulatory follicle degradation, ensuring regular egg-laying [39,40]. The GO term ‘cAMP-dependent protein kinase complex’ of cellular components was also significantly enriched. The cAMP-dependent protein kinase A (PKA) system forms a signal transduction hub during egg production, downstream of FSH signaling. It regulates gene expression by phosphorylating target transcription factors, which further affects oocyte meiosis and follicle selection [41]. The GO term ‘steroid hormone receptor activity’ of molecular functions was significantly enriched. Steroid hormone receptors are ligand-dependent transcription factors that combine with hormones to regulate the expression of target genes in different tissues. Estrogen receptors act on follicular granulosa cells, and progesterone receptors act on the smooth muscles of the oviducts, which can drive and regulate the egg production process [42].

In the KEGG enrichment analyses, the ‘PI3K-Akt signaling pathway’ and ‘PPAR signaling pathway’—which play important roles in ovarian development and egg production—showed significant enrichment. The ‘PI3K-Akt signaling pathway’ regulates the entire process of follicular development. It can be activated by FSH, which promotes granulosa cell proliferation and differentiation and enhances steroid-hormone synthesis [43]. Sustained activation of this pathway is essential for follicular recruitment, selection, and maturation. The ‘PPAR signaling pathway’ regulates steroid and lipid metabolism in the ovaries. It can indirectly participate in oocyte maturation by activating *PPARg* to regulate steroid-hormone synthesis in granulosa cells [44], promoting follicular development and maintaining optimal ovarian function.

Increasing evidence suggests that ncRNAs, such as lncRNAs, miRNAs, and circRNAs, have a wide range of functions, regulate target gene expression at multiple dimensions, and participate in the regulation of various biological activities [45,46]. The ceRNA mechanism is an important pathway through which non-coding RNAs exert their functions. Both lncRNAs [47] and circRNAs [48] can bind to miRNAs as ceRNAs and regulate gene expression. In the present study, we constructed a ceRNA network involved in the regulation of goose egg production, consisting of six lncRNAs, five miRNAs, and nine target genes. Two lncRNAs can bind miRNAs, act as ‘miRNA sponges’ and exert the function of ceRNA, including MSTRG.23007.19–miR-143-5p–*BMF* and MSTRG.40028.2–miR-205a–*AKAP1*. Studies have shown that miR-143-5p was upregulated in the pre-hierarchical follicles of Zhedong White Geese during the egg-laying stage compared to the brooding stage [49]. The expression of miR-143-5p in the ovaries of laying chickens was also higher than that in brooding chickens [50]. These findings align with the outcomes of our research, indicating that miR-143-5p may be involved in ovarian development. We found that miR-205a expression was significantly downregulated in the PE group compared to the PO group. In previous research [51], miR-205a was highly expressed in the follicles of laying hens and regulated cell apoptosis. Also, miR-205a was involved in the ceRNA regulatory network in ovarian atrophy [50]. *BMF* (BCL2 modifying factor) is a member of the B-cell lymphoma 2 family. Li et al. [52] demonstrated that the overexpression of *BMF* increased the apoptosis rate, while knocking down *BMF* significantly increased the proliferation rate of sheep granulosa cells. *BMF* can also promote the apoptosis of germ cells [53] and follicle atresia [54] in mouse ovaries. *AKAP1* (A-kinase anchoring protein 1) was upregulated during the peak egg-laying period in our study. Studies in both pigs [55] and mice [56] have shown that *AKAP1* mediated the intracellular localization of cAMP-dependent protein kinases. *AKAP1* knockout oocytes were more sensitive to the inhibitory effects of PKA elevation on meiosis, and *AKAP1* overexpression promoted meiotic resumption and maturation of oocytes.

In our ceRNA regulatory network, all the genes were differentially expressed between different egg-laying stages and participated in pathways related to egg production. The expression of miR-133a-3p was significantly upregulated in the PE and EA groups compared with that in the PR group. Zhao et al. [57] showed that miR-133a-3p negatively regulates the expression of its target gene *ANOS1* and regulates the proliferation and apoptosis of goose ovarian granulosa cells through the FGFR1 signaling pathway. miR-193a-5p was significantly downregulated in the granulosa cells of women with diminished ovarian reserve (DOR) [58], which may lead to decreased oocyte developmental ability. miR-193a-5p was also associated with the abnormal accumulation of cholesterol in the plasma membrane and the onset of polycystic ovary syndrome (PCOS) [59]. The expression of miR-375 in sexually mature ovaries of Single-Comb White Leghorn chickens was significantly lower than that in immature ovaries [60]. In addition, miR-375 was co-expressed with *CRHR1* in porcine ovarian granulosa cells and inhibited estradiol synthesis and follicular development by participating in the corticotropin-releasing hormone (CRH) signaling system [61].

*INHBB* (inhibin subunit beta B) and *A2M* (alpha-2-macroglobulin) were targeted by miR-205a. The protein encoded by *INHBB* is secreted from ovarian granulosa cells and inhibits the synthesis and release of FSH in the pituitary [62]. Decreased *INHBB* expression significantly reduced estradiol and progesterone levels [63]. Huang et al. [64] found that *INHBB* is differentially expressed in the ovaries of Ninghai indigenous chickens (high-yield) and Wuliangshan black-boned chickens (low-yield). *A2M* regulates estradiol production in ovarian granulosa cells and the development of dominant follicles [65]. This gene was differentially expressed in the ovarian medulla layers of Leizhou black ducks with high- and low-yields [66]. The present study indicated that *A2M* expression was significantly upregulated in the three comparison groups, except for PE vs. PO. *WNT9A* (Wnt family member 9A) is a ligand of the Wnt signaling pathway. This pathway mediates the development of mature granulosa cells from pre-granulosa cells and promotes oocyte growth, which is essential for maintaining ovarian function [67]. *LRP1* (LDL receptor-related protein 1) participates in the regulation of the ovarian microenvironment through tissue repairing and extracellular matrix remodeling, and can improve ovarian function in aged and premature-ovarian-failure mice [68]. Research has shown that the *LRP1* was associated with ovarian follicular development and ovulation in lambs [69]. *JAK2* (Janus kinase 2) was targeted by miR-133a-3p, MSTRG.56808.6, and MSTRG.73201.13. *JAK2* is expressed in both the nucleus and cytoplasm of oocytes and may regulate oocyte maturation by affecting the function of microfilaments during meiosis [70]. *JAK2* also regulates oocyte loss during ovarian development and influences primordial follicle formation in mice [71]. In our study, *PDGFRB* (platelet-derived growth factor receptor beta) was upregulated between PE and PR. Zhao et al. [30] also identified a higher expression level of *PDGFRB* in the ovarian tissues of Xinjiang Yili geese during the laying stage, and its mechanism of action was to promote steroid-hormone synthesis in the ovaries [72]. *MAP2K5* (mitogen-activated protein kinase kinase 5) is a key dual-specificity protein kinase member in the MAPK signaling pathway, which participates in cell proliferation, apoptosis, and reproductive processes [73]. The MAPK signaling pathway can affect folliculogenesis and egg production in ducks [74] and the differentiation and growth of chicken oviducts [75].

## 5. Conclusions

In this study, we generated mRNA, lncRNA, and miRNA expression profiles of Huoyan geese ovarian stroma tissues during the egg-laying stage through whole-transcriptome sequencing. We identified 2112 DEGs, 187 DELs, and 37 DEMs from the four different egg-laying periods. In the functional enrichment analysis, differentially expressed genes and non-coding RNAs’ target genes were enriched in multiple pathways related to ovarian development and egg production processes, such as ‘ovulation cycle’ and ‘PI3K-Akt signaling pathway’. Through the target relationship prediction between DELs, DEMs, and DEGs, we ultimately constructed a ceRNA regulatory network related to egg-laying traits, which included six lncRNAs, five miRNAs, and nine genes. These results provide information for exploring molecular regulatory mechanisms and accelerating the genetic improvement of egg production performance in geese.

## Figures and Tables

**Table 1:** The number of upregulated and downregulated DEGs, DELs, and DEMs.

	Upregulated Genes	Downregulated Genes	Upregulated lncRNAs	Downregulated lncRNAs	Upregulated miRNAs	Downregulated miRNAs
PE vs. PR	1142	263	56	30	19	3
PE vs. EA	782	330	41	29	14	5
PE vs. PO	89	107	26	14	3	1
EA vs. PR	225	13	23	9	6	2

## Data Availability

The whole-transcriptome sequencing data of total RNA were submitted to the NCBI Sequence Read Archive (SRA) with accession number BioProject: PRJNA1400528.

## Supplementary Materials

The following supporting information can be downloaded at: https://www.mdpi.com/article/10.3390/ani16071053/s1, Table S1: Primer sequences for qRT-PCR; Table S2: Summary of strand-specific library RNA sequencing data; Table S3: Summary of small RNA sequencing data; Table S4: List of differentially expressed genes; Table S5: List of differentially expressed lncRNAs; Table S6: List of differentially expressed miRNAs; Table S7: List of cis- and trans-target genes of DELs; Table S8: List of target genes of DEMs; Table S9: List of significantly enriched GO terms; Table S10: List of significantly enriched KEGG pathways.

## Abbreviations

The following abbreviations are used in this manuscript:

ceRNA	Competitive endogenous RNA
DEGs	Differentially expressed genes
DELs	Differentially expressed lncRNAs
DEMs	Differentially expressed miRNAs
GO	Gene Ontology
KEGG	Kyoto Encyclopedia of Genes and Genomes
