# Supplementary material for: Whole-Transcriptome Sequencing Integrative Analyses Reveal Expression Profiles and ceRNA Regulatory Network of Huoyan Goose Egg Production
Source: Animals (Basel). 2026 Mar 30;16(7):1053. doi: 10.3390/ani16071053 (PMC13072419; doi:10.3390/ani16071053)
Supplement: Supplementary file 1 [file animals-16-01053-s001.zip › Table S2_Summary of strand-specific library RNA sequencing data.docx]

**Table S2: Summary of strand-specific library RNA sequencing data**

| **Samples** | **Clean data** | **GC** | **Q30** | **Total reads** | **Mapped reads** | **Mapped ratio** |
| --- | --- | --- | --- | --- | --- | --- |
| **early-1** | 17,945,216,158 | 45.86 | 92.71 | 120,116,236 | 111,977,393 | 93.22% |
| **early-2** | 15,397,611,026 | 45.99 | 92.25 | 103,184,522 | 95,917,624 | 92.96% |
| **early-3** | 15,805,228,364 | 46.17 | 91.55 | 105,698,350 | 98,831,526 | 93.50% |
| **early-4** | 16,410,814,896 | 46.07 | 91.06 | 109,859,052 | 102,354,202 | 93.17% |
| **early-5** | 16,660,562,664 | 44.85 | 94.01 | 111,550,364 | 104,782,889 | 93.93% |
| **peak-1** | 15,909,960,030 | 44.50 | 94.46 | 106,465,870 | 98,909,904 | 92.90% |
| **peak-2** | 18,147,540,874 | 44.91 | 93.82 | 121,423,412 | 112,651,894 | 92.78% |
| **peak-3** | 17,075,899,444 | 44.51 | 93.75 | 114,279,530 | 106,168,341 | 92.90% |
| **peak-4** | 15,143,618,098 | 44.84 | 94.32 | 101,340,730 | 94,861,729 | 93.61% |
| **peak-5** | 16,197,632,588 | 44.44 | 94.47 | 108,389,164 | 100,742,594 | 92.95% |
| **post-1** | 16,081,195,320 | 44.78 | 92.04 | 107,627,012 | 100,107,203 | 93.01% |
| **post-2** | 16,616,032,374 | 44.67 | 92.42 | 111,226,190 | 103,228,697 | 92.81% |
| **post-3** | 17,522,443,822 | 44.46 | 92.25 | 117,164,530 | 108,870,590 | 92.92% |
| **post-4** | 15,595,900,328 | 45.17 | 90.59 | 104,364,860 | 96,787,784 | 92.74% |
| **post-5** | 17,957,639,028 | 45.05 | 92.94 | 120,224,002 | 112,009,231 | 93.17% |
| **pre-1** | 17,954,673,730 | 45.55 | 92.63 | 120,190,932 | 112,614,367 | 93.70% |
| **pre-2** | 17,120,951,008 | 44.57 | 91.87 | 114,595,660 | 106,367,740 | 92.82% |
| **pre-3** | 16,396,409,446 | 44.42 | 92.89 | 109,740,876 | 102,278,173 | 93.20% |
| **pre-4** | 16,226,420,170 | 45.29 | 92.20 | 108,568,302 | 101,082,519 | 93.11% |
| **pre-5** | 18,065,754,056 | 45.65 | 93.77 | 120,806,900 | 114,141,694 | 94.48% |

Note: Clean data, total base number (Gb); GC (%), percentage of guanine and cytosine bases in the total nucleotides; Q30 (%), percentage of bases with values greater than or equal to Q30 in the clean data; Total reads, number of clean reads; Mapped reads, number of reads mapped to the reference genome among clean reads; Mapped ratio, percentage of reads mapped to the reference genome among clean reads.
