# Supplementary material for: Whole-Transcriptome Sequencing Integrative Analyses Reveal Expression Profiles and ceRNA Regulatory Network of Huoyan Goose Egg Production
Source: Animals (Basel). 2026 Mar 30;16(7):1053. doi: 10.3390/ani16071053 (PMC13072419; doi:10.3390/ani16071053)
Supplement: Supplementary file 1 [file animals-16-01053-s001.zip › Table S3_Summary of small RNA sequencing data.docx]

| **Samples** | **Raw reads** | **Length<18 and >30** | **Low quality** | **Containing**  **'N' reads** | **Clean reads** | **Q30(%)** |
| --- | --- | --- | --- | --- | --- | --- |
| **early-1** | 10,271,964 | 483,436 | 16 | 8 | 9,788,504 | 96.21 |
| **early-2** | 10,298,231 | 544,612 | 18 | 4 | 9,753,597 | 96.71 |
| **early-3** | 10,322,210 | 721,410 | 24 | 8 | 9,600,768 | 97.80 |
| **early-4** | 10,069,717 | 457,306 | 27 | 8 | 9,612,376 | 97.82 |
| **early-5** | 10,008,745 | 388,112 | 12 | 7 | 9,620,614 | 96.89 |
| **peak-1** | 9,871,202 | 329,949 | 6 | 5 | 9,541,242 | 96.63 |
| **peak-2** | 15,072,574 | 771,229 | 25 | 10 | 14,301,310 | 97.49 |
| **peak-3** | 13,007,131 | 344,664 | 10 | 5 | 12,662,452 | 98.14 |
| **peak-4** | 10,228,182 | 483,534 | 24 | 3 | 9,744,621 | 96.65 |
| **peak-5** | 12,769,696 | 382,077 | 14 | 0 | 12,387,605 | 97.49 |
| **post-1** | 10,075,658 | 363,853 | 13 | 1 | 9,711,791 | 97.07 |
| **post-2** | 14,423,321 | 494,121 | 15 | 0 | 13,929,185 | 97.01 |
| **post-3** | 13,457,088 | 222,151 | 9 | 0 | 13,234,928 | 94.53 |
| **post-4** | 9,991,478 | 202,109 | 5 | 12 | 9,789,352 | 96.22 |
| **post-5** | 13,955,184 | 360,660 | 15 | 5 | 13,594,504 | 96.51 |
| **pre-1** | 11,099,578 | 951,234 | 20 | 0 | 10,148,324 | 96.34 |
| **pre-2** | 10,343,906 | 563,056 | 11 | 1 | 9,780,838 | 98.17 |
| **pre-3** | 12,735,465 | 479,816 | 15 | 0 | 12,255,634 | 97.06 |
| **pre-4** | 12,564,773 | 772,568 | 13 | 0 | 11,792,192 | 98.22 |
| **pre-5** | 16,581,807 | 961,585 | 48 | 3 | 15,620,171 | 95.85 |

**Table S3 Summary of small RNA sequencing data**

Note: Raw reads, raw sequencing data; Length<18 and >30, the number of reads with a length less than 18 and more than 30 nucleotides after removing adapters; Low quality, the number of reads with more than 20% of bases having a quality score below 30; Containing 'N' reads, the number of reads containing at least 10% of unknown bases 'N'; Q30 (%), percentage of bases with values greater than or equal to Q30 in the clean data.
